# Endoscopic dacryocystorhinostomy: A comparison of double-flap and single-flap techniques

**DOI:** 10.1016/j.amsu.2020.03.005

**Published:** 2020-04-08

**Authors:** Majid Bani-Ata, Abdelwahab Aleshawi, Muayyad Ahmad, Omar Saleh, Raneem Ashour, Hanen Khalil, Safwan Alomari, Ala”a A. Alhowary

**Affiliations:** a Otolaryngology Branch, Department of Special Surgery, Jordan University of Science and Technology, P.O Box 3030, Irbid, 22110, Jordan; b Intern, King Abdullah Hospital, Irbid, 22110, Jordan; c Clinical Nursing Department, School of Nursing, University of Jordan, Amman, 11942, Jordan; d Ophthalmology Branch, Department of Special Surgery, Faculty of Medicine, Jordan University of Science and Technology, P.O Box 3030, Irbid, 22110, Jordan; e Department of Anesthesia, Jordan University of Science and Technology, P.O Box 3030, Irbid, 22110, Jordan

**Keywords:** Endoscopic, Dacryocystorhinostomy, Mucosal flap, Epiphora, Recurrence

## Abstract

**Background:**

Dacryocystorhinostomy (DCR) is a procedure to restore the flow of tears into the nose from the lacrimal sac when the nasolacrimal duct obstructed. This study aimed to compare the success rates of two different techniques in endonasal endoscopic DCR; namely single and double mucosal flap techniques.

**Material and methods:**

A nonequivalent quasi-experiment design was used in this study. Retrospectively, patients underwent endoscopic DCR for primary nasolacrimal duct (NLD) obstruction were included. Patients were divided into the single-flap technique and the double-flap technique groups. Success was defined as the achievement of patency of the NLD throughout the period of follow-up with significant improvement in epiphora.

**Results:**

Overall, 77 cases were included in the final analysis. Mean age was 41 years and 60% were female. Forty-six cases underwent the single-flap technique and 31 cases underwent the double-flap technique. Recurrence of NLD obstruction occurred in 11 (23.9%) cases in the single-flap group and in only one case (3.2%) in the double-flap group.

**Conclusion:**

The modified double-flap technique for primary NLD obstruction resulted in less recurrence compared to the single-flap technique. Creating double flaps to cover any exposed lacrimal bone may reduce the rate of postoperative adhesions over the nasolacrimal duct ostium.

## Highlights

• DCR is used to restore the tears into the nose from the obstructed lacrimal sac.
• Single and double mucosal flap techniques can be used.
• Double-flap technique for primary NLD obstruction resulted in less recurrence.

## 1 Introduction

Dacryocystorhinostomy (DCR) is a surgical bypass procedure that creates an anastomosis between the lacrimal sac and the nasal mucosa via a bony ostium. It is commonly indicated in cases of nasolacrimal duct (NLD) obstruction. It can be performed externally through a skin incision or internally through the nasal cavity with or without endoscopic visualization [1].

Many reports have comparatively studied the external and endonasal DCR approaches. Most have demonstrated that despite the advantages and disadvantages inherent in each approach, both can be considered acceptable alternatives as they had similar success rates and surgical outcomes with minimal complications [[2], [3], [4], [5]]. Despite endoscopic DCR being more commonly practiced nowadays, only a few studies have compared the success rates for different surgical techniques used in this procedure.

## 2 Material and Methods

A nonequivalent quasi-experiment design was used in this study. Patients were divided into two comparison groups based on the surgical technique used: a single-flap technique versus a double-flap technique. Retrospectively, data collected from charts included patient demographics, surgical notes, and postoperative success rates.

We received approval from the institutional review board at our university-based tertiary hospital to conduct the study. In addition, permission was granted to review data of patients who underwent endoscopic DCR for primary NLD obstruction, between January 2016 and December 2018. In addition, this study was registered into the Research Registry and conducted in accordance with the declaration Helsinki.

Eligibility criteria included all patients who had epiphora with or without purulent eye discharge. The diagnosis of NLD obstruction was confirmed by the ophthalmologist. Males and females, regardless of age, were included in the study. Exclusion criteria included incomplete medical records, follow up period of less than 1 year postoperatively, history of previous DCR surgery, and the indication for the DCR not being primary NLD obstruction; including secondary obstructions due to trauma or radiation, lacrimal sac abscesses, or nasolacrimal infections.

The diagnosis of NLD obstruction was confirmed by the ophthalmologist by probing and irrigation and the exclusion of the other eye disease that may result in similar symptoms. After removal of the DCR tube, success was defined as the achievement of patency of the NLD throughout the period of follow up as confirmed by probing and irrigation. Furthermore, the patient reporting the disappearance of epiphora. The recurrence of NLD obstruction was documented when the patient reported epiphora again with the ophthalmologist verifying obstruction of the NLD by probing and irrigation. Endoscopic DCR was performed in all patients with the collaboration between an ENT surgeon and an ophthalmologist at the hospital.

The surgeons started with a single flap technique and noticed the recurrence rate is relatively high (almost quarter of the cases); the decision was taken to do the surgery for the other group with the double flap technique.

**Fig. 1:**
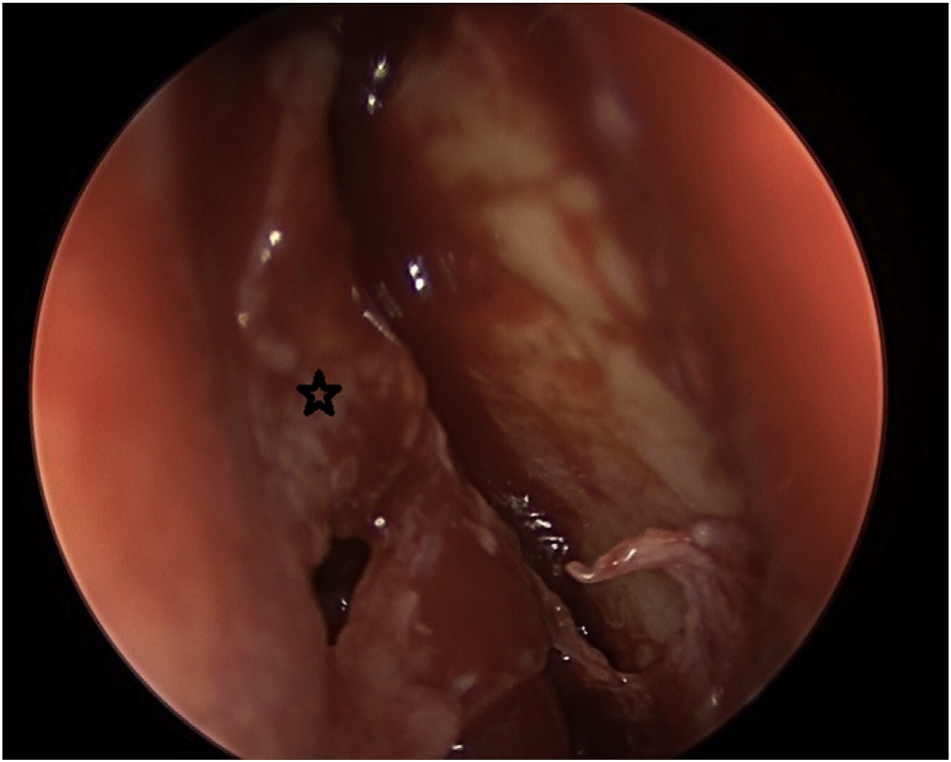
The first flap consists of the lateral nasal wall mucosa covering the lacrimal bone (black star).

**Fig. 2:**
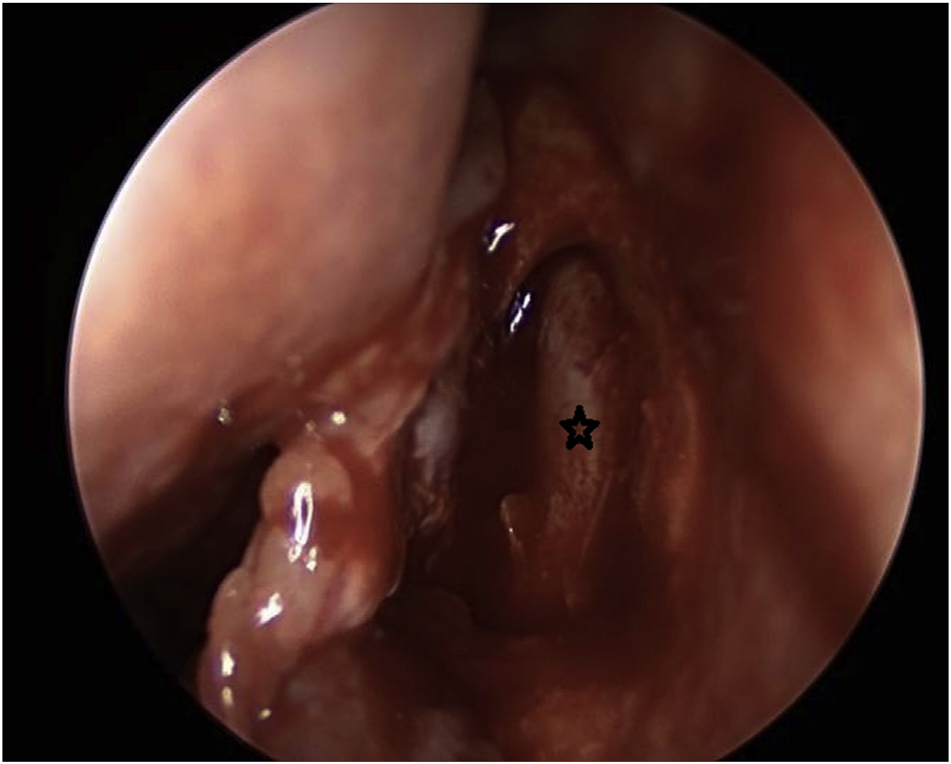
Exposed lacrimal sacafter removing the lacrimal bone (black star).

**Fig. 3:**
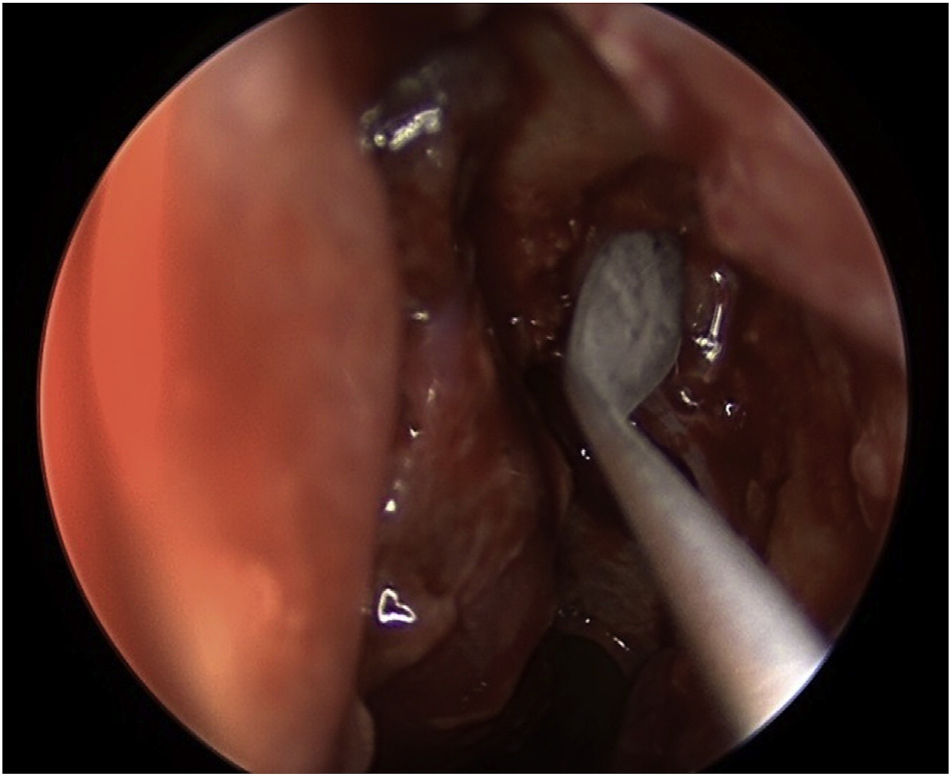
The second flap is created in the lacrimal sac using a keratotomy knife.

The procedure for the single-mucosal flap technique included raising up the nasal mucosal flap above the axilla of the middle turbinate by 8–10 mm until a junction between the lacrimal bone and the frontal process of the maxilla is established (Fig. 1). Next step is to remove the lacrimal bone and expose the entire lacrimal sac. From this point, a Kerrison type Hajek Koeffler is used to complete the removal of the more resistant bone portion of the lacrimal fossa of the maxilla, with the assistance of the drill over the thick part of the bone, without damaging the lacrimal sac (Fig. 2). Then, the ophthalmologist starts in dilatation of the upper and lower puncta and insertion a probe until touching the exposed lacrimal sac. At this point, a longitudinal incision is made in the lacrimal sac by a keratotomy knife (Fig. 3). After the DCR tube has been inserted by the ophthalmologist, part of the mucosal flap is resected at the site of the DCR tube exit [7]. The remaining part of the mucosal flap is repositioned to the lateral wall to ensure not to keep any exposed bone, and the upper part of the mucosal flap can be repositioned on the axilla of the middle turbinate, when applicable, to cover any remaining bone to this level. In the double flap technique, the surgeon proceeds with the same steps done in the single-flap technique by meaning of elevating the lateral mucosal flap, removing the lacrimal bone, exposing the entire lacrimal sac and creating a longitudinal incision in the sac and trimming part of the mucosal flap at the exit site of the DCR tube. The procedure to be added in the double-flap technique is to create a second flap in the lacrimal sac.

**Fig. 4:**
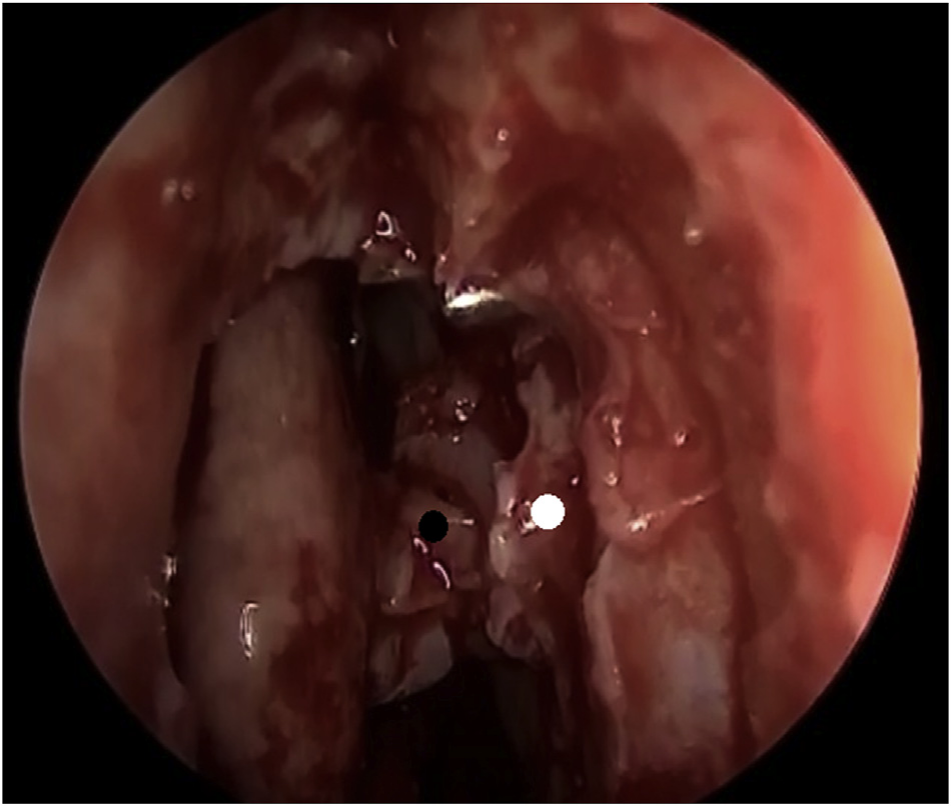
The lacrimal sac flap is opened as a “window” by reflecting the posterior part posteriorly (black dot) and the anterior part anteriorly (white dot).

**Fig. 5:**
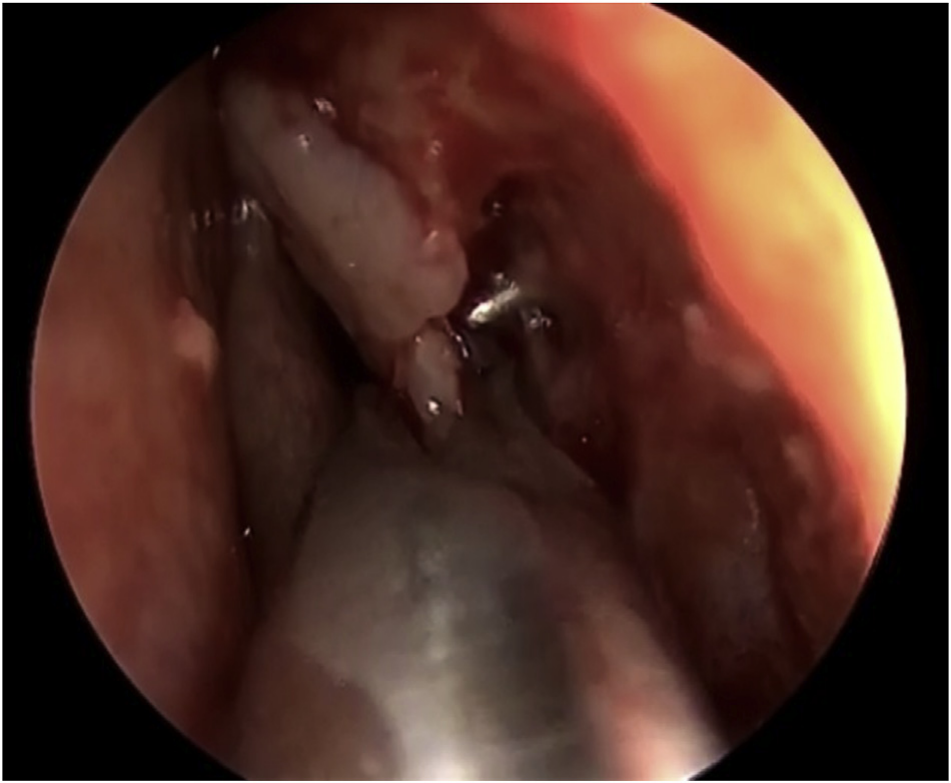
Trimming of the mucosal flap.

**Fig. 6:**
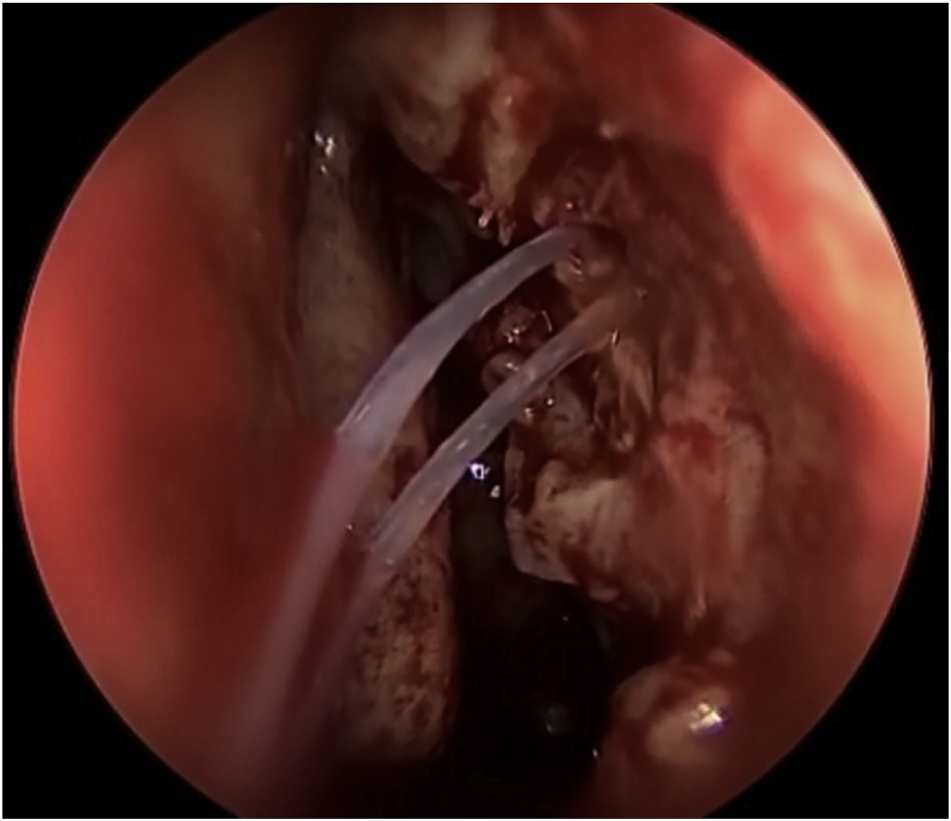
Repositioned flaps.

The second flap is elevated from the lacrimal sac at the site of the DCR tube exit after the previously made longitudinal incision in the lacrimal sac, then, two incisions are made anteriorly, and two incisions are made posteriorly. The posterior flap of the sac is reflected posteriorly, and the anterior flap is reflected anteriorly to achieve marsupialization of the lacrimal sac into the nasal cavity (Fig. 4). The posterior flap of the lacrimal sac will be in direct contact with the mucosal flap initially made (Fig. 5). The upper part of the mucosal flap can be repositioned on the axilla of the middle turbinate, when applicable, to cover any remaining bone to this level. The mucosa that covers the wall of the agger nasi can also be juxtaposed to the medial wall of the lacrimal sac flap (Fig. 6). The flaps are finally fixed by applying gel-foam.

All patients were discharged home on the same day of surgery with prophylactic oral antibiotic and without steroid nasal spray. The first follow up visit was scheduled at 1 week after surgery. At the 3-month visit the DCR tube was removed at the outpatient clinic. For cases who had a recurrence of NLD obstruction, revision surgery was scheduled and the NLD tube was kept in place for at least 6 months postoperatively.

Using Chi-Square test to calculate sample size based on alpha level = 0.05, power = 0.80, and a moderate effect size = 0.33, the minimum needed sample size was 72 cases [6]. The sample size in this study was 77 cases. The study was conducted at a university-based tertiary center over 5 years, between January 2016 and December 2018.

## 3 Results

The mean age for the 77 participants was 41.6 years (SD = 20.5), 52 of them were female (67.5%). The mean time for the 12 cases who had a recurrence or follow up was 21.6 months (SD = 8.4). The single-flap technique group had 46 patients (60%) with a mean age of 40.3 years (SD = 23.4) and a mean follow up of 22.2 months (SD = 8.5). The double-flap technique group had 31 patients with a mean age of 43.3 years (SD = 15.4) and the mean time for follow up at 20.7 months (Table 1).

**Table 1:** Descriptive statistics for the study variables (N = 77).

Variable	N	%
Age (Mean = 41.56, SD = 20.51)		
Range (1–80 years)		
Gender		
Male	25	32.5
Female	52	67.5
Flap side		
Right	36	46.8
Left	41	53.2
Flap number		
Single	46	59.7
Double	31	40.3
Recurrence		
No	65	84.4
Yes	12	16.6

Recurrence of NLD obstruction occurred in eleven cases (23.9%) in the single-flap group compared to one case (3.2%) in the double-flap group (Fisher's Exact Test = 6.024, p = 0.022). The 12 patients from both groups who had a recurrence of the condition underwent revision surgery. 3 patients (27.3%) from the single-flap had a recurrence of the condition for the second time. The patient in the double flap did not have a second recurrence of the condition. There were no significant differences in age (p = 0.818), gender (p = 0.128), or the side of the flap (p = 0.814) among groups (Table 2).

**Table 2:** Comparison on single versus double flap with sample characteristics.

	Flap				Test statistics χ^2^	p-value
	Single		Double			
Gender		%		%	2.313	.128
Male	18	72.0	7	28.0		
Female	28	53.8	24	46.2		
Recurrence						
No	35	53.8	30	46.2	Fisher's Exact Test = 6.024	.022
Yes	11	91.7	1	8.3		
Flap side						
Right	21	58.3	15	41.7	.056	.814
Left	25	61.0	16	39.0		
Age						
1-46	24	58.5	17	41.5	.053	.818
47-80	22	61.1	14	38.9		

## 4 Discussion

The first endonasal endoscopic technique used in our study is described as a single mucosal flap technique, where a lateral wall mucosal flap is elevated to expose the lacrimal sac by removing the lacrimal bone. With the ophthalmologist passing the DCR tube, the lacrimal sac is opened longitudinally without creating a flap within the lacrimal wall.

In contrast, endoscopic DCR by the double flap technique is based on creating two flaps. In addition to elevating the mucosal flap over the lacrimal bone, an additional flap in the lacrimal sac is created. Both flaps can be used to cover any exposed bone after the passage of the DCR tube by the ophthalmologist. The upper part of the mucosal flap can be repositioned on the axilla of the middle turbinate and the mucosa that covers the wall of the agger nasi will also be juxtaposed to the medial wall of the lacrimal sac flap. It is believed that with the use of such measures, leaving any exposed bone can be minimized or avoided, therefore improving postoperative healing [8]. Since re-closure of the nasolacrimal stoma is commonly due to aberrant healing with granulation tissue and synechiae after endoscopic DCR [9], it seems probable that mucosa-sparing techniques may offer better success rates.

Another important benefit of the double flap technique is enhancing better healing at the stoma site and prevention of granulation tissue and epithelialization. Thus, this may lead to a reduction of the chance of blockage of the new stoma, which explains the higher success rate of the double flap technique versus the single flap technique. In addition, the double flap technique enhances a better ostium opening.

Indeed, in our double-flap cohort, where we attempted to cover any exposed bone by mucosal tissue during surgery, the posterior flap of the lacrimal sac will be in direct contact with the mucosal flap initially made, and excessive healing reactions may have been minimized or avoided, that will guarantee a good newly formed orifice for the DCR, resulting in a significantly lowered recurrence rate compared to the single-flap cohort.

Mucosa-sparing techniques in endoscopic DCR have been recommended in other studies. A modification to a technique described by Tsirbas and Wormald in which the nasal mucosa is preserved and brought in contact with the lacrimal mucosa has been described [10]. The authors report that leaving an epithelialized surgical site at the end of the operation may prevent the closure of the ostium and leads to a high success rate comparable with that of external DCR [11]. Peng et al. reported another modified preserved nasal and lacrimal mucosal flap technique that was simple and safe and offers effective coverage of the bare bone around the opened sac and provided a similar or even better clinical outcome compared with the other routine treatment techniques used for NLD obstruction [12]. Similarly, another recent article emphasized the importance of minimizing bone exposure in endoscopic DCR by describing a novel bi-pedicled interlacing mucosa-sparing flap technique. They report 100% anatomical patency rate in 55 patients [13].

Although the benefits of mucosal preservation and bone coating endoscopic DCR have also been demonstrated in systematic reviews, studies that have a cohort with mucosa-sparing technique in direct comparison with other cohorts are in shortage. In a large meta-analysis, a systematic review of randomized clinical trials with evidence relating to the preservation of mucosal flaps in DCR surgery between 1970 and 2015 was conducted. After applying the acceptance criteria, two randomized control trials and three comparative studies, with the best available evidence being at level 1B, were included in analysis. The authors conclude that there is a trend towards improved outcomes and reduced granulation in groups where nasal mucosal and lacrimal flaps were preserved. However, this may not be clear-cut evidence as only two studies have shown a statistically significant benefit of such a technique [9].

To the best of our knowledge, our current study is the first to directly compare the success rate of two distinct techniques with different utilization of the nasal or lacrimal mucosa in endoscopic DCR for NLD obstruction. We have found that maximizing coverage of any bare-bone by utilizing the double-flap technique where two mucosal flaps, one nasal and one lacrimal, are created and used, has a clear benefit over the classical single-flap technique which utilizes one nasal flap only. This superiority was reflected by the significantly higher success rate in the double-flap cohort compared to the single-flap cohort in our report.

Limitations to our study include lack of randomization of the patients to the two treatment arms. Nonetheless, several points of strength in our study can be mentioned including a standardized diagnostic approach for detection of NLD patency or obstruction including objective (by probing and irrigation) and subjective (symptoms including epiphora) methods, standardized surgeon, surgical settings, and techniques in endoscopic DCR, extended follow up for the patients of at least 12 months after surgery to detect any recurrence of the condition and a reasonably high number of cases in our cohorts.

## 5 Conclusion

Creating double flaps to cover any exposed lacrimal bone may reduce the rate of postoperative adhesions over the nasolacrimal duct ostium. A larger body of evidence on the clinical outcomes and complication rates of different methods used in endoscopic DCR is needed. Larger and controlled randomized trials that compare these approaches as well as studies of innovation and novel techniques that may advance our progress in treating primary NLD obstruction and improve results in our patients, are warranted.

This study was conducted according to the Strengthening the reporting of cohort studies in surgery (STROCSS) 2019 Guideline [14].

## Ethical approval

Institutional approval was obtained from the Institutional Review Board at Jordan University of Science and Technology.

## Sources of funding

No funding.

## Author contribution

All authors contributed significantly and in agreement with the content of the article. All authors were involved in project design, data collection, analysis, statistical analysis, data interpretation and writing the manuscript. All authors presented substantial contributions to the article and participated of correction and final approval of the version to be submitted.

## Research registration number

Researchregistry5383

https://www.researchregistry.com/browse-the-registry#home/registrationdetails/5e56a2752ffc78001562b9e6/checked 27/02/20.

## Guarantor

Dr. Majid Bani-Ata.

## Data availability

The datasets generated and analyzed during the current study are available from the corresponding author.

## Provenance and peer review

Not commissioned externally peer reviewed.

## Ethics and patient consent

Written informed consent was obtained from the patients for publication. Institutional approval was obtained from the Institutional Review Board at Jordan University of Science and Technology. This study was conducted in accordance with the Declaration of Helsinki.

## Declaration of competing interest

The authors declare that they have no competing interests.
